# Neural Responses to Reward in a Gambling Task: Sex Differences and Individual Variation in Reward-Driven Impulsivity

**DOI:** 10.1093/texcom/tgaa025

**Published:** 2020-06-19

**Authors:** Guangfei Li, Sheng Zhang, Thang M Le, Xiaoying Tang, Chiang-Shan R Li

**Affiliations:** Department of Psychiatry, Yale University School of Medicine, New Haven, CT 06519, USA; Department of Biomedical Engineering, School of Life Sciences, Beijing Institute of Technology, Beijing 10081, China; Department of Psychiatry, Yale University School of Medicine, New Haven, CT 06519, USA; Department of Psychiatry, Yale University School of Medicine, New Haven, CT 06519, USA; Department of Biomedical Engineering, School of Life Sciences, Beijing Institute of Technology, Beijing 10081, China; Department of Psychiatry, Yale University School of Medicine, New Haven, CT 06519, USA; Department of Neuroscience, Yale University School of Medicine, New Haven, CT 06511, USA; Interdepartmental Neuroscience Program, Yale University School of Medicine, New Haven, CT 06511, USA

**Keywords:** delay discounting, fMRI, gender, HCP, punishment

## Abstract

Previous work suggests sex differences in reward sensitivity. However, it remains unclear how men and women differ in the neural processes of reward-driven impulsivity. With a data set of 968 subjects (502 women) curated from the Human Connectome Project, we investigated sex differences in regional activations to reward and to punishment in a gambling task. Individual variations in reward-driven impulsivity were quantified by the difference in reaction time between reward and punishment blocks in the gambling task, as well as by a behavioral measure of delay discounting. At a corrected threshold, men and women exhibited significant differences in regional activations to reward and to punishment. Longer reaction times during reward versus punishment blocks, indicative of more cautious responding, were associated with left-hemispheric lateral prefrontal cortical activation to reward in men but not women. Steeper discounting was associated with higher activation to reward in the right-hemispheric dorsal anterior cingulate cortex and angular gyrus in women but not men. These sex differences were confirmed in slope tests. Together, the results highlight the sex-specific neural processes of reward-driven impulsivity with left-hemispheric prefrontal cortex supporting impulse control in men and right-hemispheric saliency circuit playing a more important role in diminished impulse control in women.

## Introduction

Individuals vary in how they respond to reward-related contingencies, and a literature accumulates to focus on sex differences in reward processing. For instance, men showed higher reward sensitivity and/or sensation-seeking in questionnaire assessment (Li et al. 2007a; Barch et al. 2013; Eneva et al. 2017). In a recent work of a reward go/no-go task, men exhibited greater physiological arousal to go responses (predominating monetary wins), which was also more predictive of go success rate, relative to women (Le et al. 2019). A meta-analysis showed that men were more risk taking, as evidenced in laboratory decision-making tasks as well as self-reported and observed behaviors (Byrnes et al. 1999). However, other studies suggested a more complex picture (Reynolds et al. 2006; Beck and Triplett 2009). An earlier review reported sex differences favoring females most consistently for interpersonal social tasks (e.g., control of emotions), less consistently for behavioral control tasks (e.g., delayed gratification), and inconsistently for other cognitive challenges (e.g., conceptual tempo) (Bjorklund and Kipp 1996). Authors suggested that sex differences in impulse control are domain-specific, with women demonstrating greater abilities on tasks related to reproduction and childrearing, in accord with the parental investment theory (Bjorklund and Kipp 1996). Other reviews demonstrated more consistent sex differences in self-control in children, with females showing an advantage prior to the onset of puberty, and mixed results in adults (Hosseini-Kamkar and Morton 2014; Weinstein and Dannon 2015).

Previous studies have associated delay discounting (DD), a construct of reward-related impulsivity that reflects how quickly or steeply individuals discount the value of a future reward, with reward sensitivity (Appelhans et al. 2011), impulsivity or poor inhibitory control (Evenden and Ryan 1996; Kirby et al. 1999), and risk-taking (Booth et al. 1999). Animal studies have addressed sex differences in DD. Male rats chose immediate over delayed larger rewards more frequently than females (Bayless et al. 2013). On the other hand, in a study to explore the roles of dopaminergic signaling in DD, Eubig and colleagues reported no sex difference in percent choices at baseline, whereas d-amphetamine administration led to <80% larger-reinforcer choice in half of the female but none of the male animals (Eubig et al. 2014). In humans, a meta-analysis showed that women were better at delaying gratification than men, and the sex differences held consistently across age groups (Silverman 2003). However, later findings appeared to be mixed, with some studies reporting larger DD rates in females (Reynolds et al. 2006; Beck and Triplett 2009), others in males (Kris and Nino 1996; Peper et al. 2013; Shibata 2013) or no differences (de Wit et al. 2007; Lucas and Koff 2010; Cross et al. 2011). In other studies, men showed steeper discounting than women only when challenged concurrently with a working memory task (Mei et al. 2017), testosterone concentrations were correlated with DD rates positively and negatively, respectively, in females and males (Doi et al. 2015), and alcohol-dependent men but not women discounted delayed gains more steeply, in contrast to their controls (Myerson et al. 2015).

Together, it remains unclear whether or how men and women differ in reward sensitivity and reward-driven impulse control, and the findings may depend on clinical versus laboratory evaluations, specific experimental manipulations, and subject populations.

With functional magnetic resonance imaging (fMRI) or electroencephalography, investigators have reported sex differences in neural responses to reward and punishment. For instance, in a study combining electroencephalography and a guessing task with reward or punishment feedback, boys showed lower feedback-related negativity and less changes in postpunishment behavior (Ding et al. 2017). Further, only girls demonstrated feedback-related negativity to monetary punishment in relation to a reward sensitivity trait. In a sample of 190 subjects from the Human Connectome Project (HCP), women relative to men showed greater suppression of the default mode network and higher activation of the dorsal attention network to both reward and punishment, suggesting enhanced saliency response in women (Dumais et al. 2018).

Many studies characterized the neural correlates of reward sensitivity and sensation-seeking, with a specific focus on the ventral striatum (VS; see Richards et al. 2013; Telzer 2016 for a review) and VS reward response as a marker of depression, attention deficit hyperactivity disorder (ADHD), and drug or behavioral addiction (Luking et al. 2016; Clark et al. 2019; Noda et al. 2020). For instance, visual sexual stimuli activated the VS in both sexes, but the VS was involved in the distractor effects of sexual stimuli on performance in line orientation judgment in men only (Strahler et al. 2018). In risk-related decision-making, boys relative to girls showed higher VS activation, as mediated by greater motivation to earn money (Alarcon et al. 2017). A recent work showed that endotoxin (vs. placebo, to induce neuro-inflammation, as may occur during chronic stress) led to decreased VS activity in anticipation of reward in female but not male participants (Moieni et al. 2019), suggesting sex differences in contextual modulation of reward responses. A meta-analysis revealed a medium effect size of VS hypo-responsiveness in children and adults with ADHD, whereas VS reward response correlated positively with impulsivity scores in the healthy population (Plichta and Scheres 2014).

Thus, imaging studies have revealed sex differences in neural response to a multitude of reward-related constructs in health and illness. On the other hand, no studies to our knowledge have specifically examined how men and women may differ in the neural processes of individual variation in reward-driving impulsivity. The current study aimed to fill this gap of research. Specifically, we employed a data set curated from the HCP to examine the sex-specific neural correlates of individual variation in reward-driven impulsivity. The HCP comprised an imaging dataset collected of a gambling task (Delgado et al. 2000), where participants guessed at the identity of a card to win money in alternating largely-rewarding and largely-punishing blocks. We quantified the reaction time (RT) difference between the reward and punishment blocks as a measure of impulsivity; a prolonged RT during reward versus punishment blocks suggests more cautious responding and impulse control (Kareken 2019). Participants in the HCP were also evaluated with a DD task outside the scanner, which provided an additional measure of reward-driven impulsivity. We analyzed the data of men and women together as well as separately in linear regressions against these behavioral indices. When sex-specific correlates were identified, we computed the regional activities (parameter estimates) for all subjects and confirmed or refuted the sex differences with slope tests as in our previous studies (Ide et al. 2020; Jenks et al. 2020; Li et al. 2020).

## Materials and Methods

### Dataset

For the present study, we have obtained permission from the HCP to use both the Open and Restricted Access data. The data of a total of 968 adults (502 women; 29.6 ± 3.6 years of age, mean ± SD) were included (Table 1). All subjects were physically healthy with no severe neurodevelopmental, neuropsychiatric, or neurologic disorders. Subject recruitment procedures and informed consents, including consent to share deidentified data, were approved by the Washington University Institutional Review Board.

**Table 1:** Demographics and behavioral measures of men and women

Characteristic	Men (*n* = 466)	Women (*n* = 502)	*P* value^^*^^
Age (years)	27.9 ± 3.6	29.6 ± 3.6	0.000
Education (years)	14.8 ± 1.9	15.0 ± 1.8	0.132
AUC$200	0.275 ± 0.217	0.256 ± 0.191	0.079
AUC$40K	0.542 ± 0.282	0.514 ± 0.283	0.403
AUC$40K—AUC$200	0.249 ± 0.211	0.258 ± 0.216	0.566
RT_REW (ms)	404.8 ± 118.4	432.6 ± 106.4	0.000
RT_PUN (ms)	382.6 ± 114.4	417.2 ± 108.6	0.000
RT_REW-RT_PUN (ms)	19.7 ± 53.2	14.5 ± 54.2	0.159

Note: Values are mean ± SD; AUC, area under the curve; RT_REW: RT of reward block; RT_PUN: RT of punishment block. The RT data were missing in a total of 96 subjects (30 women) from the HCP; thus, 872 subjects (472 women) were included in the RT statistics.

^^*^^Two-sample *t*-test with age and years of education as covariates.

All participants were assessed with a DD task (DDT). DD describes the undervaluing of rewards that are delayed in time. The version of the DDT employed in the HCP identified the “indifference points” at which subjects were equally likely to choose a larger reward later (e.g., $200 in 3 years) versus a smaller reward sooner (e.g., $100). An adjusting-amount approach was used on the basis of Green and Myerson’s work (Estle et al. 2006; Green et al. 2007), in which delays were fixed and reward amounts were adjusted trial by trial based on subjects’ choices. In brief, 2 initial reward amounts ($200 and $40K) and 6 fixed delays (1, 6 months, 1, 3, 5 and 10 years) were used in the DDT. An area under the curve (AUC) measure provided an index of how steeply an individual discounted delayed rewards (Myerson et al. 2001). The AUC is the sum of 6 area of trapezoids; for each trapezoid, the area was equal to (*x*_2_ − *x*_1_) ([*y*_1_ + *y*_2_]/2), where *x*_1_ and *x*_2_ were delays, and *y*_1_ and *y*_2_ were the subjective values associated with these delays. All *x* and *y* values were normalized via division by the largest *x* and *y* value, respectively, so the AUC ranged from 0 (maximal discounting) to 1 (no discounting), with a smaller AUC reflecting greater discounting and impulsivity (Reynolds 2006).

Investigators have observed a magnitude effect in the DDT, with larger amounts discounted less steeply than smaller amounts (Green et al. 2013). That is, the extent of discounting decreases with the amount and tends to level off as the amount of delayed reward becomes very large. Across a wide range of monetary amounts ($20–$10 million), it was noted that the steepest change of discounting occurs between $100 and $50K (Green et al. 2013). As individuals differ in socioeconomic status and may perceive the value of $200 and $40K differently, we employed the difference in AUC for the 2 amounts—AUC$40K—AUC$200—as a measure of reward-driven impulsivity in data analyses.

### Gambling Task for fMRI

The gambling task was adapted from a paradigm developed by Delgado and colleagues (Delgado et al. 2000). Each subject completed 2 runs of the task each with 4 blocks—2 of punishment and 2 of reward—in a fixed order (run 1: punishment—reward—punishment—reward; and run 2: reward—punishment—punishment—reward) with a fixation period (15 s) between blocks. The participants were asked to guess whether the number of a mystery card (represented by a “?” and ranging from 1 to 9) was larger or smaller than 5 by pressing a corresponding button (Barch et al. 2013). The feedbacks comprised a green up-pointing arrow for correct guess and $1 win, a red down-pointing arrow for $0.5 loss; or a gray double-headed arrow for a wash (when the mystery card number was 5). The mystery number was controlled by the program and shown for 1.5 s, followed by the feedback for 1.0 s. There was a 1.0 s intertrial interval with a “+” shown on the screen. Each block contained 8 trials. In reward blocks, 6 win trials were pseudorandomly interleaved with either 1 neutral and 1 loss trial, 2 neutral trials, or 2 loss trials. In punishment blocks, 6 loss trials were interleaved with either 1 neutral and 1 reward trial, 2 neutral trials, or 2 reward trials. Thus, the amount of money won was the same across subjects.

### Imaging Protocol and Data Preprocessing

MRI was done using a customized 3T Siemens Connectome Skyra with a standard 32-channel Siemens receiver head coil and a body transmission coil. T1-weighted high-resolution structural images were acquired using a 3D MPRAGE sequence with 0.7 mm isotropic resolution (FOV = 224 × 224 mm, matrix = 320 × 320, 256 sagittal slices, TR = 2400 ms, TE = 2.14 ms, TI = 1000 ms, FA = 8°) and used to register functional MRI data to a standard brain space. FMRI data were collected using gradient-echo echo-planar imaging (EPI) with 2.0 mm isotropic resolution (FOV = 208 × 180 mm, matrix = 104 × 90, 72 slices, TR = 720 ms, TE = 33.1 ms, FA = 52°, multiband factor = 8, 253 frames, ~3 m and 12 s/run).

We inspected and removed individuals’ data deemed of poor quality. Further, the gamble task data were missing for 126 subjects. As a result, a total of 968 out of 1206 subjects were included in the current study.

Imaging data were analyzed with Statistical Parametric Mapping (SPM8, Welcome Department of Imaging Neuroscience, University College London, UK), following our published routines (Wang et al. 2019; Zhang et al. 2019; Zhornitsky et al. 2019). Standard image preprocessing was performed. Images of each individual subject were first realigned (motion corrected). A mean functional image volume was constructed for each subject per run from the realigned image volumes. These mean images were coregistered with the high-resolution structural MPRAGE image and then segmented for normalization with affine registration followed by nonlinear transformation. The normalization parameters determined for the structural volume were then applied to the corresponding functional image volumes for each subject. Finally, the images were smoothed with a Gaussian kernel of 4 mm at full width at half maximum.

### Imaging Data Modeling

We modeled the BOLD signals to identify regional brain responses to punishment block versus baseline and reward block versus baseline. A statistical analytical block design was constructed for each individual subject, using a general linear model with a boxcar each for punishment or reward blocks convolved with a canonical hemodynamic response function (HRF). Realignment parameters in all 6 dimensions were entered in the model as covariates. Serial autocorrelation caused by aliased cardiovascular and respiratory effects was corrected by a first-degree autoregressive model. The general linear model estimated the component of variance that could be explained by each of the regressors. In the first-level analysis, we constructed for individual subjects a statistical contrast of reward block versus baseline, punishment block versus baseline, as well as reward versus punishment block to evaluate brain regions that responded to wins and losses and that responded differently to wins and losses. The contrast images (difference in β) of the first-level analysis were then used for the second-level group statistics.

In group analyses, we conducted a one-sample *t*-test to identify regional responses to reward versus baseline, punishment versus baseline, and reward versus punishment for men and women together and separately. We compared men and women in two-sample *t*-tests of the same contrasts with age and years of education as covariates to evaluate sex differences in regional responses.

To examine how regional brain responses to these contrasts varied with individual differences in DD, we conducted a whole-brain multiple regression on reward versus baseline and on punishment versus baseline against “AUC$40K—AUC$200” in men and women together, with age, sex and years of education as covariates, and in men and women separately, with age and years of education as covariates. In addition to DD, the gambling task provided RT data during reward and punishment blocks. We derive “RT_reward—RT_punishment” as an additional measure of impulsivity; a longer RT during reward versus punishment blocks suggests more cautious responses and impulse control. As the latter behavioral index was derived by contrasting reward and punishment blocks, we performed whole-brain linear regression of the contrast “reward versus punishment” against “RT_reward—RT_punishment” in men and women combined, with age, sex and years of education as covariates, as well as in men and women separately, with age and years of education as covariates.

Following current reporting standards, all imaging results were evaluated with voxel *P* < 0.001, uncorrected, in combination with a cluster *P* < 0.05, corrected for family-wise error (FWE) of multiple comparisons, on the basis of Gaussian random field theory, as implemented in SPM (Poldrack et al. 2008). All voxel activations were reported in Montreal Neurological Institute (MNI) coordinates and neurological orientation.

In ROI analysis, we used MarsBar (http://marsbar.sourceforge.net/) to derive for each individual subject the activity (β contrast, averaged across voxels) of the ROIs. Functional ROIs were defined based on clusters obtained from whole-brain analysis. For ROIs identified from linear regressions in men or women alone, we tested sex differences directly with a slope test, with age and years of education as covariates and showed two-tailed *P* values (Zar 1999).

## Results

### D‌D and Gambling Task Performance

**Figure 1:**
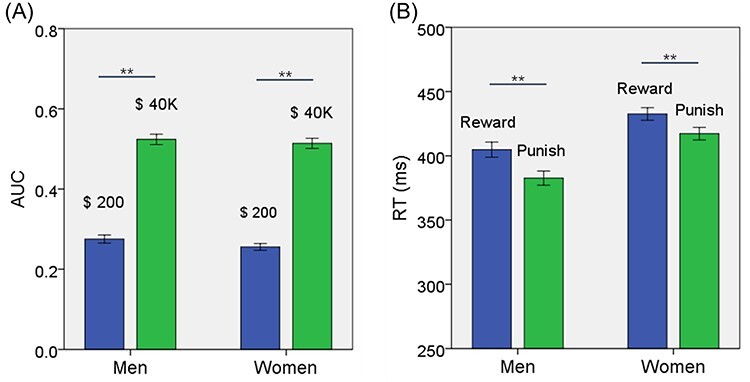
(*A*) The values (mean ± SE) of AUC$200 and AUC$40K shown separately for men and women. AUC$40K was larger than AUC$200 for both men and women. (*B*) The RT (mean ± SE) for reward and punishment blocks shown separately for men and women. Reward blocks showed slower RT than punishment blocks for both men and women. ^*^^*^P < 0.001. See text for details of statistics.

For measures of DD—AUC$200 and AUC$40K—an analysis of variance (ANOVA) with sex (men vs. women) as a between-subject variable and amount ($200 vs. $40K) as a within-subject variable showed a significant amount main effect (*F* = 1362.698, *P* < 0.001), but not sex main effect (*F* = 1.063, *P* = 0.303), or sex × amount interaction (*F* = 0.505, *P* = 0.478). Post hoc analyses showed that AUC$200 was smaller than AUC$40K in men and women combined (*t* = −36.976, *P* < 0.001; paired-sample *t* test), in men (*t* = −25.475, *P* < 0.001), and in women (*t* = −26.788, *P* < 0.001) (Fig. 1*A*). These findings confirmed the magnitude effect and showed no sex differences in DD. Men and women did not show differences in AUC$200, AUC$4K, or AUC$4K—AUC$200 (Table 1).

For RT, we performed an ANOVA with sex (men vs. women) as a between-subject variable and block type (reward vs. punishment) as a within-subject variable. The results showed a significant main effect of sex (*F* = 16.087, *P* < 0.001) and block type (*F* = 88.076, *P* < 0.001), but not sex × block type interaction (*F* = 2.028, *P* = 0.155). In post hoc analyses, both men (*t* = 7.420, *P* < 0.001) and women (*t* = 5.828, *P* < 0.001) showed slower RT during reward than punishment blocks (Fig. 1*B*). Men relative to women showed faster RT in both reward (*t* = −3.658, *P* < 0.001) and punishment (*t* = −4.704, *P* < 0.001) blocks (Table 1).

### Brain Activations to Reward and Punishment

We examined regional responses to reward versus null (baseline) blocks in a one-sample *t* test of the entire cohort and of men and women separately. Supplementary Figure 1*A*–*C* show the results.

**Figure 2:**
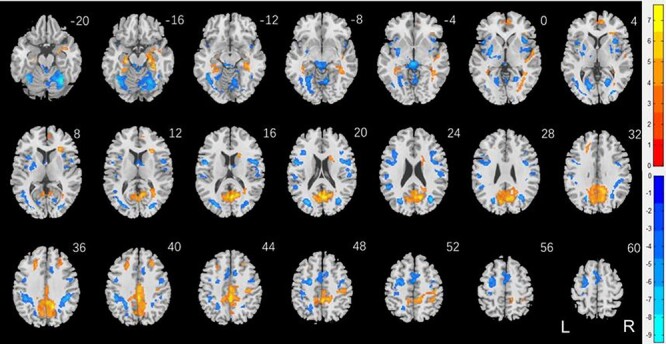
Sex differences in regional responses to reward: two-sample *T* test of the contrast (reward-baseline) between men and women with age and years of education as covariates. Voxel *P* < 0.001, uncorrected. All clusters with cluster *P* < 0.05, corrected for FWE of multiple comparisons, are shown in Table 2. Color bars show voxel *T* values; warm: men > women, cool: women > men. Clusters are overlaid on a T1 structural image in neurological orientation: right = right.

To examine sex differences, we conducted a two-sample *t* test to compare men and women with age and years of education as covariates. At voxel *P* < 0.001, uncorrected, in combination with cluster-level *P* < 0.05, FWE-corrected, men relative to women showed higher activation in midline visual cortex, including the calcarine sulcus, precuneus, posterior cingulate cortex, superior parietal cortex, bilateral anterior superior frontal gyrus (SFG), parahippocampal gyrus, and frontopolar cortex (Fig. 2). Women relative to men showed higher activation in bilateral cerebellum, thalamus in the region of the habenula, dorsomedial prefrontal cortex in the supplementary motor area, multiple loci in bilateral lateral occipital cortex and temporal parietal cortex, left posterior SFG, bilateral putamen, and right anterior insula. These clusters are summarized in Table 2.

**Table 2:** Sex differences in regional responses to reward

Region	Cluster size (*k*)	Peak voxel (*Z*)	Cluster FWE *P*-value	MNI coordinates (mm)		
				*X*	*Y*	*Z*
Men > women						
Cingulum_Mid_L	4366	7.62	0.000	0	--38	44
ParaHippocampal_R	737	6.64	0.000	24	--22	--16
Frontal_Mid_L	208	5.37	0.000	--20	28	36
Temporal_Sup_R	131	5.31	0.001	56	--2	0
Frontal_Sup_Medial_R	149	4.99	0.001	10	52	2
Frontal_Mid_R	124	4.96	0.002	28	28	42
Caudate_R	93	4.80	0.009	18	18	16
Women > men						
Cerebelum_6_R	1262	Inf	0.000	32	--58	--20
Cerebelum_6_L	1639	7.60	0.000	--36	--62	--20
Precentral_L	1881	6.62	0.000	-50	2	18
Rolandic_Oper_R	690	6.33	0.000	50	4	16
Lingual_R	154	6.29	0.000	20	--58	2
Putamen_L	551	6.10	0.000	-30	-6	-2
Supp_Motor_Area_L	732	5.79	0.000	0	-4	54
Insula_R	192	5.78	0.000	38	14	2
SupraMarginal_L	137	5.39	0.001	--52	--46	24
SupraMarginal_R	218	4.83	0.000	54	--22	20
R insula^*^	175	4.68	0.000	38	0	2

Note: Brain regions were identified by reference to the Automated Anatomic Labeling or AAL Atlas (Tzourio-Mazoyer et al. 2002). We referred to Duvernoy’s atlas (Duvernoy 2009) for coordinates (^*^) that were not identified by the AAL. R: right; L: left.

We showed one-sample *t* test results of punishment versus baseline for men and women combined as well as separately in Supplementary Figure 2*A*–*C*.

**Figure 3:**
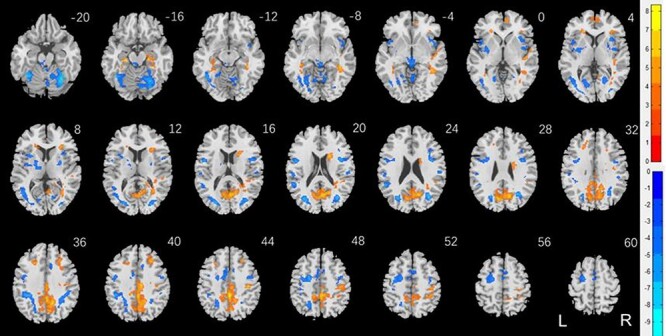
Sex differences in regional responses to punishment: two-sample *T* test of the contrast (punishment-baseline) between men and women with age and years of education as covariates. Voxel *P* < 0.001, uncorrected. All clusters with cluster *P* < 0.05, corrected for FWE of multiple comparisons, are shown in Table 3. Color bars showed voxel *T* values; warm: men > women, cool: women > men. Clusters are overlaid on a T1 structural image in neurological orientation: right = right.

Figure 3 shows the results of two-sample *t* test of men versus women, with years of age and of education as covariates, on punishment versus baseline. The clusters are summarized in Table 3. Men relative to women showed higher activation in the occipital cortex, including the calcarine sulcus, precuneus, posterior cingulate cortex, superior parietal cortex, bilateral anterior SFG, bilateral parahippocamal gyrus, right caudate head, and frontopolar cortex. Women relative to men showed higher activation in bilateral cerebellum, thalamus in the region of the habenula, bilateral putamen and insula, bilateral posterior SFG and middle frontal cortex, and dorsomedial prefrontal cortex in the supplementary motor area.

**Table 3:** Sex differences in regional responses to punishment

Region	Cluster size (*k*)	Peak voxel (*Z*)	Cluster FWE *P*-value	MNI coordinates (mm)		
				*X*	*Y*	*Z*
Men > women						
Cingulum_Mid_R	3816	Inf	0.000	2	--38	44
Caudate_R	336	6.38	0.000	18	10	20
ParaHippocampal_L	201	6.17	0.000	--24	--24	--16
R Mid Temporal C^*^	315	5.99	0.000	40	--42	--6
Temporal_Sup_R	491	5.54	0.000	46	--16	2
Frontal_Mid_R	207	5.22	0.000	28	28	42
Frontal_Med_Orb_R	120	4.86	0.003	8	52	0
Women > men						
Cerebelum_6_R	1069	Inf	0.000	34	--56	--22
Fusiform_L	2201	7.69	0.000	--38	--52	--22
R Lat Occipital C^*^	660	7.20	0.000	34	--68	22
Thalamus^*^	258	6.30	0.000	2	--32	--6
R Mid/Inf Frontal C^*^	311	6.17	0.000	40	4	22
Rolandic_Oper_R	690	6.33	0.000	50	4	16
Rolandic_Oper_L	1301	6.09	0.000	--48	2	18
Insula_R	349	5.24	0.000	34	16	6
Insula_L	443	5.03	0.000	--34	12	8
Lingual_R	87	4.85	0.015	20	--60	4
SupraMarginal_R	67	4.54	0.047	54	--22	20
SupraMarginal_L	92	4.10	0.012	--56	--24	20

Note: Brain regions were identified by reference to the Automated Anatomic Labeling or AAL Atlas (Tzourio-Mazoyer et al. 2002). We referred to Duvernoy’s atlas (Duvernoy 2009) for coordinates (^*^) that were not identified by the AAL. R: right; L: left; Lat: lateral; Mid: middle; Inf: inferior; C: cortex.

### Brain Activations in Relation to Individual Variation in DD

**Figure 4:**
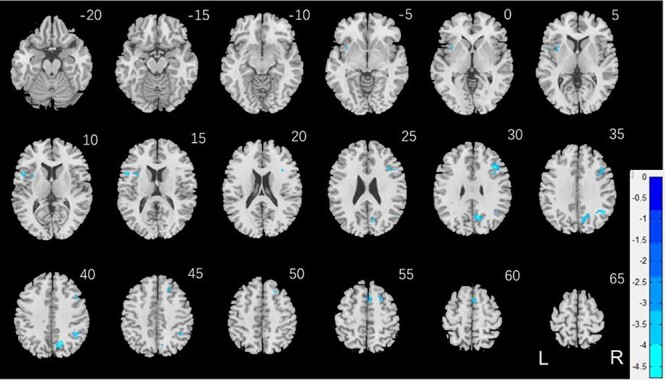
Regional responses of reward versus baseline in correlation with “AUC$40K—AUC$200” across all subjects. Bilateral inferior frontal cortex, right precuneus, right SFG, R AG, left anterior insula, and presupplementary motor area showed higher activation in negative correlation with “AUC$40K—AUC$200” (Table 4).

We conducted a whole brain linear regression of the contrast “reward versus baseline” against “AUC$40K—AUC$200” across all subjects, with sex, age and years of education as covariates. At the threshold of voxel *P* < 0.001, uncorrected, in combination with cluster *P* < 0.05 FWE-corrected, the results showed clusters in negative correlation with “AUC$40K—AUC$200” in bilateral inferior frontal cortex, right precuneus, right angular gyrus, right SFG, left anterior insula, and presupplementary motor area (Fig. 4). Thus, higher activation of these regions was associated with a smaller “AUC$40K—AUC$200” or greater reward-driven impulsivity. The clusters are summarized in Table 4.

**Table 4:** Regional responses of reward versus baseline in correlation with “AUC$40K—AUC$200” across all subjects

Region	Cluster size	Peak voxel (*Z*)	Cluster FWE *P*-value	MNI coordinates (mm)		
				*X*	*Y*	*Z*
Negative						
Frontal_Inf_Tri_R	195	4.73	0.000	38	24	30
Angular_R	151	4.48	0.000	44	-56	38
Precuneus_R	242	4.47	0.000	14	-64	40
Frontal_Inf_Oper_L	60	4.28	0.040	-52	14	16
Frontal_Sup_R	72	4.14	0.018	20	30	44
Supp_Motor_Area_R	77	4.10	0.013	2	12	58
Insula_L	100	4.07	0.003	-34	10	16

Note: Brain regions were identified by reference to the Automated Anatomic Labeling or AAL Atlas (Tzourio-Mazoyer et al. 2002). R: right; L: left.

Likewise, we conducted a whole brain linear regression of the contrast “punishment versus baseline” against “AUC$40K—AUC$200” across all subjects, with sex, age and years of education as covariates. No clusters showed significant correlations at the same threshold.

**Figure 5:**
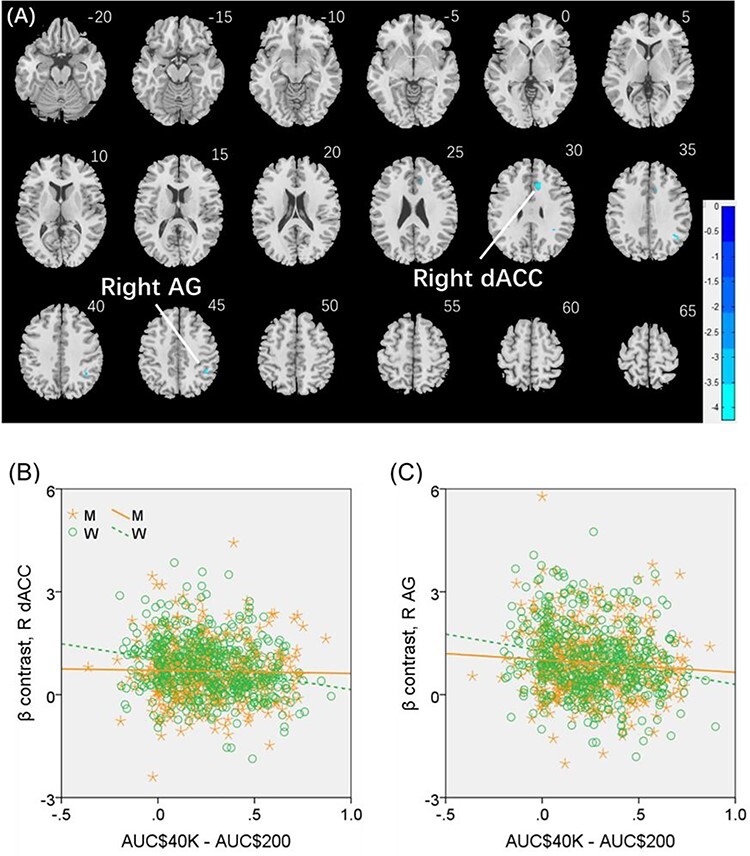
(*A*) Regional responses of reward versus baseline in correlation with “AUC$40K—AUC$200” across women in the R dACC and R AG. (*B* and *C*) Region of interest analyses to confirm sex differences in the correlation. Each data point represents 1 subject. Orange/green color: men (M)/women (W). Slope tests show sex differences in the regressions of the R dACC (*Z* = 3.3, *P* = 0.001) and R AG (*Z* = 2.03, *P* = 0.0424).

We conducted the same analysis separately for men and for women both with age and years of education as covariates. For reward versus baseline, no clusters showed significant correlations in men. In women, a cluster in the right dorsal anterior cingulate cortex (R dACC: *x* = 10, *y* = 22, *z* = 30, volume = 704 mm^3^, *Z* = 4.20) and in the right angular gyrus (R AG: *x* = 42, *y* = −46, *z* = 46, volume = 648 mm^3^, *Z* = 4.00) showed significant negative correlation with “AUC$40K—AUC$200”. These 2 clusters are shown in Figure 5*A*. For punishment versus baseline, no clusters showed significant correlation with “AUC$40K—AUC$200” in men or in women.

We extracted for individual subjects the parameter estimate (β contrast) of reward versus baseline for the R dACC and R AG and tested for sex differences in the regression. The results showed R dACC responses in significant negative correlation with “AUC$40K—AUC$200” in women (*r* = −0.224, *P* < 0.001), as expected, but not in men (*r* = −0.015, *P* = 0.754). The sex difference was confirmed in a slope test (*Z* = 3.3, *P* = 0.001; Fig. 5*B*). Likewise, the R AG showed response in significant negative correlation with “AUC$40K—AUC$200” in women (*r* = −0.215, *P* < 0.001), as expected, but not in men (*r* = −0.087, *P* = 0.062). The sex difference was also confirmed by the slope test (*Z* = 2.03, *P* = 0.0424; Fig. 5*C*). Notably, compared with men, women showed a marginally higher β contrast value for the R dACC (men: 0.69 ± 0.82; women: 0.81 ± 0.87; *P* = 0.030; two-sample *t*-test with age and years of education as covariates) but not for the R AG (men: 0.93 ± 1.02; women: 0.95 ± 1.02; *P* = 0.280).

### Brain Activations in Relation to Individual Variation in RT during Reward versus Punishment Blocks

We showed one-sample *t* test results of reward versus punishment for men and women combined as well as separately in Supplementary Figure 3*A*–*C*. We conducted a two-sample *t* test of men versus women for “reward—punishment” and no clusters showed significant differences at voxel *P* < 0.001, uncorrected, in combination with cluster *P* < 0.05 FWE-corrected. For both men and women, reward relative to punishment blocks engaged higher activation of the ventral striatum, bilateral caudate head, ventromedial prefrontal cortex, posterior cingulate cortex/precuneus, and midline occipital cortex.

We conducted a whole brain linear regression of the contrast “reward versus punishment” against “RT_reward—RT_punishment” across all subjects, with sex, age and years of education as covariates. As the RT of a total of 96 subjects (30 women) were not recorded in the raw data of HCP, 872 subjects (472 women) were included in the analysis. At the threshold of voxel *P* < 0.001, uncorrected, in combination with cluster *P* < 0.05 FWE-corrected, the result showed a cluster in positive correlation with “RT_reward—RT_punishment” in the left SFG or L SFG (*x* = −28, *y* = −2, *z* = 52, volume = 856 mm^3^, *Z* = 4.37).

**Figure 6:**
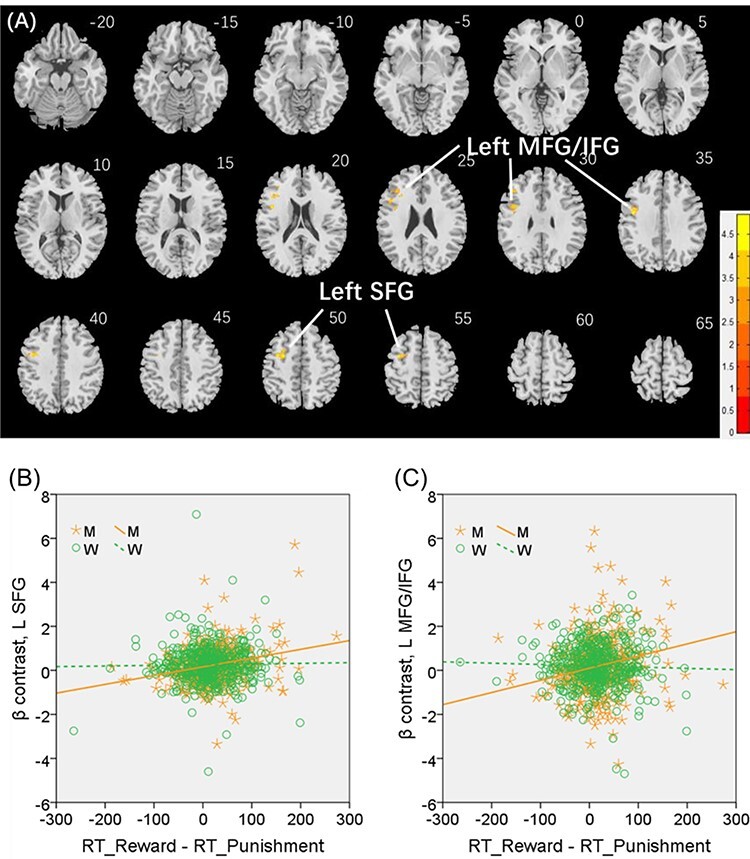
(*A*) Regional responses of reward versus punishment in correlation with “RT_reward—RT_punishment” (ms) across men in the left SFG (L SFG) and left middle/inferior frontal gyrus (L MFG/IFG). (*B* and *C*) Region of interest analyses to confirm sex differences in the regressions of the L SFG (*Z* = 3.56, *P* = 0.0004) and L MFG/IFG (*Z* = 3.37, *P* = 0.0008), respectively, with slope tests.

We conducted the same analysis separately for men and for women both with age and years of education as covariates. A cluster in the L SFG (*x* = −28, *y* = −2, *z* = 52, volume = 3320 mm^3^, *Z* = 4.87), at the same coordinate of the cluster identified for men and women combined, and another in the left middle/inferior frontal gyrus (L MFG/IFG: *x* = −44, *y* = 30, *z* = 30, volume = 496 mm^3^, *Z* = 3.85) showed significant positive correlation with “RT_reward—RT_punishment” in men (Fig. 6*A*). No clusters showed significant correlations in women.

We extracted the parameter estimate (β contrast) for these men-specific correlates of reward versus punishment—the L SFG and L MFG/IFG—for all subjects and tested for sex differences in the regression. The results showed L SFG responses in significant positive correlation with “RT_reward—RT_punishment” in men (*r* = 0.260, *P* < 0.001), as expected, but not in women (*r* = 0.023, *P* = 0.614). The sex difference was confirmed in a slope test (*Z* = 3.56, *P* = 0.0004; Fig. 6*B*). Likewise, the L MFG/IFG showed activities in significant positive correlation with “RT_reward—RT_punishment” in men (*r* = 0.203, *P* < 0.001), as expected, but not in women (*r* = −0.024, *P* = 0.611). The sex difference was also confirmed by the slope test (*Z* = 3.37, *P* = 0.0008; Fig. 6*C*). Notably, men and women did not differ in the β contrast values of reward versus punishment for the L SFG (men: 0.24 ± 0.82; women: 0.26 ± 0.84; *P* = 0.564) or the L MFG/IFG (men: 0.21 ± 1.44; women: 0.20 ± 1.17; *P* = 0.884).

## Discussion

Using the HCP data, we evaluated sex differences in regional responses to reward and to punishment and sex-specific correlates of reward-driven impulsivity, as reflected in the RT difference between reward and punishment blocks in the gambling task and DD for $40K versus $200. Steeper discounting, as shown in a smaller AUC$40K—AUC$200, was associated with higher activation of the right-hemispheric dorsal anterior cingulate cortex (dACC) and angular gyrus (AG) in women but not in men. Longer RTs during reward versus punishment blocks, indicative of more cautious responding, were associated with higher activation of left-hemispheric lateral prefrontal cortex (lPFC), encompassing the superior/middle/inferior frontal gyrus, in men but not in women. The sex differences were all confirmed in slope tests. Together, these results highlight the sex-specific neural processes of individual variation in reward-driven impulsivity with left-hemispheric lPFC supporting impulse control in men and right-hemispheric saliency circuit mediating diminished impulse control in women.

### Sex Differences in Regional Activations to Reward and to Punishment

Compared with women, men showed higher or lower activation to reward and to punishment in many of the same brain areas, such that a direct contrast revealed no significant sex differences in regional activities for “reward versus punishment.” Areas showing higher activation in men included the medial occipital cortex, precuneus, posterior cingulate cortex, superior parietal cortex, bilateral anterior SFG, bilateral parahippocampal gyrus, and frontopolar cortex. Areas showing higher activation in women included bilateral cerebellum, thalamus in the region of the habenula, dorsomedial prefrontal cortex in the supplementary motor area, left posterior SFG, bilateral putamen, and right anterior insula. While we would not attempt to offer a post hoc account of the sex differences in these areal activations, it seems that, for both responses to reward and to punishment, men engage areas supporting memory, visual attention and self-control (Hu et al. 2016) whereas women engage the saliency circuit, including the thalamus and insula, to a greater extent (Hendrick et al. 2010).

Previous studies have examined sex differences in regional brain activations in the Iowa gambling task (Bolla et al. 2004; Overman et al. 2006; van den Bos et al. 2013; Singh 2016), DD task (McClure et al. 2004), and other paradigms that involved evaluation of reward against risk (Sidlauskaite et al. 2018). In contrast, in the gambling task of the HCP, participants guessed at the card number, and the decision-making process involved in card guessing were likely different from those implicated in the Iowa gambling or DD task. The regional activities may reflect primarily the responses to feedback and/or the congruency/incongruency between guessed and true card identity, with most guesses correct and incorrect, respectively, in reward and punishment blocks. Indeed, for both men and women, reward relative to punishment blocks engaged higher activity of the ventral striatum and medial orbitofrontal cortex, areas known to process rewarding stimuli (Diekhof et al. 2012; Oldham et al. 2018; Wang et al. 2020).

### Sex Differences in Individual Variation in Reward-Driven Impulsivity

Using RT difference between reward and punishment blocks in the gambling task and DD for $40K versus $200, we demonstrated sex-specific correlates of impulse control. Steeper discounting, as shown in a smaller AUC$40K—AUC$200, was associated with higher activation of the right-hemispheric dACC and AG in women but not in men. The dACC and AG are each an important hub of the saliency and ventral attention system (Behrens et al. 2007; Zhang et al. 2012; Ide et al. 2013; Mueller-Pfeiffer et al. 2014; Manza et al. 2016; Leuchs et al. 2017). It would seem that diminished impulse control is associated with higher attentional responses to reward in women.

Both men and women displayed prolonged RT in reward as compared with punishment blocks, suggesting individuals’ belief/ownership and manifestation of self-control when their actions led to desired outcomes. Longer RTs during reward versus punishment blocks, indicative of more cautious responding, were associated with higher activation of left-hemispheric lPFC, in the superior/middle/inferior frontal gyri, in men but not in women. The left lPFC plays important roles in cognitive control (Farr et al. 2012; Zhang and Li 2012). An earlier study showed that functional disruption of the left, but not right, lPFC with low-frequency repetitive transcranial magnetic stimulation increased choices of immediate over larger delayed rewards (Figner et al. 2010). Repetitive transcranial magnetic stimulation did not change choices involving only delayed rewards or valuation judgments of immediate versus delayed rewards, suggesting a specific role of the left lPFC in the control of intertemporal choice. In support, a recent meta-analysis associated diminution of left lPFC activity with less preference for delayed choices (Schuller et al. 2019). Interestingly, a structural imaging study demonstrated lower volumes in the left lPFC in individuals with higher reward sensitivity, as assessed with the Punishment and Sensitivity to Reward Questionnaire (Adrian-Ventura et al. 2019). Thus, the current findings add to this literature by specifying a specific role of the left lPFC in underscoring individual variation in reward-driven impulse control in men.

### Potential Clinical Implications

Notably, the correlations of these regional activities with behavioral measures exhibited sex differences, as confirmed by slope tests. These findings suggest sex-specific neural processes that underlie individual variation in reward-driven impulsivity and may have implications for elucidating the biomarkers of neuropsychiatric conditions. For instance, girls but not boys with ADHD showed steeper DD, as compared with their controls (Patros et al. 2018; Rosch et al. 2018). Considering the current findings, one may speculate that heightened saliency response to reward may, at its extreme, be associated with ADHD pathology in girls more so than in boys. In contrast, functional near-infrared spectroscopy of mostly male children (>70%) with ADHD demonstrated diminished left lPFC activation during inhibitory control in a go/no-go task (Miao et al. 2017). A magnetoencephalography study demonstrated diminished gamma band activity in the left lPFC in children with ADHD (75% male) during time estimation (Wilson et al. 2013). In another predominantly male (>85%) sample, older children with ADHD were able to compensate for deficits in interference control in the Stroop task by increasing left lPFC activation (Yasumura et al. 2019). The potential of the left lPFC dysfunction as an etiological marker was also supported by spectroscopy findings of lower choline ratios in the left prefrontal regions in medication-naïve children with ADHD (Benamor 2014) and morphometric findings of diminished left PFC convolution complexities in boys with ADHD (Li et al. 2007a, 2007b), relative to controls.

Thus, if the current findings of sex differences in neurotypical populations replicate in individuals with ADHD, ADHD pathology may be driven by excessive right-hemispheric bottom-up, saliency processes in females and by dysfunctional left-hemispheric top-down, control processes in males. This hypothesis can be tested with a behavoral paradigm that clearly distinguishes these neural processes.

### Limitations of the Study and Related Issues

A number of limitations need to be considered for the study. First, the HCP did not contain imaging data collected of the DD task. Therefore, although we evaluated how individual variation in DD related to reward responses in the gambling task, it remains unclear how the differences in DD may translate to cerebral responses to intertemporal choice. Second, the HCP focused on neurotypical populations. Although a substance use disorder that required treatment was an exclusion criterion, some participants used alcohol, nicotine, and/or cannabis, and more men than women were substance users. Thus, it remains unclear how the current findings may reflect the consequences of substance use. Third, men and women did not exhibit differences in regional activations to reward versus punishment. However, one needs to consider the block design of the gambling task as well as the admixture of both win and loss trials (though disproportionately) in reward and punishment blocks. This experimental design may have masked sex differences that could be revealed in event-related studies, as well as other psychological constructs, including prediction error (Cao et al. 2019) and post feedback behavioral adjustment (Ding et al. 2017), which may vary with individual reward sensitivity. Further, we demonstrated the neural correlates in relation to reward but not punishment. However, the psychological processes of reward and punishment are interrelated (Le et al. 2020), and studies that allow a clear distinction between reward and punishment feedback are needed to evaluate how individual differences in impulse control may manifest in regional responses to punishment. Fourth, impulsivity is a multidimensional construct. The current findings addressed reward-driven impulsivity, whereas sex differences in attentional and motor impulsivity remain to be investigated in future work (Ray Li et al. 2005; Farr et al. 2012). Further, the current findings should be considered as specific to monetary reward, and more studies are needed to address how men and women differ in behavioral and neural sensitivity to other types (e.g., social) of reward (Spreckelmeyer et al. 2009; Greimel et al. 2018). Finally, reward sensitivity and neural responses to reward appeared to vary significantly with age (Schreuders et al. 2018; Dhingra et al. 2020). Thus, the current findings should be considered as specific to the age range (young adulthood) of the current sample.

## Conclusion

Using a large public domain data set, we identified the sex-specific correlates of reward-driven impulsivity. Individual variation in impulsivity was reflected by regional activations of the dACC and R AG—higher saliency responses to reward may influence impulse control—in women. Individual variation in impulsivity was reflected by regional activation of the left-hemispheric lPFC—diminished behavioral control may play a more important role in impulsivity—in men.

## Notes

Data were provided by the Human Connectome Project, WU-Minn Consortium (Principal Investigators: David Van Essen and Kamil Ugurbil; 1U54MH091657) funded by the 16 National Institutes of Health (NIH) Institutes and Centers that support the NIH Blueprint for Neuroscience Research; and by the McDonnell Center for Systems Neuroscience at Washington University. The current study used data collected by the NIH Human Connectome Project. Processed data and computer codes used in data analyses will be available upon request to investigators for nonprofit research. All subject recruitment procedures and informed consents, including consent to share deidentified data, were approved by the Washington University Institutional Review Board. *Conflict of Interest*: None declared.

## Funding

National Institutes of Health (grants MH113134, DA023248, DA045189, AG067024, AA021449 to C.-S.R.L.); a scholarship from the China Scholarship Council for G.L. to visit Yale University.

## Supplementary Material

Li_etal_HCP_Reward-Impulsivity_Supplement_tgaa025
