# Supplementary material for: Neural Responses to Reward in a Gambling Task: Sex Differences and Individual Variation in Reward-Driven Impulsivity
Source: Cereb Cortex Commun. 2020 Jun 19;1(1):tgaa025. doi: 10.1093/texcom/tgaa025 (PMC7446303; doi:10.1093/texcom/tgaa025)
Supplement: Li_etal_HCP_Reward-Impulsivity_Supplement_tgaa025 [file li_etal_hcp_reward-impulsivity_supplement_tgaa025.docx]

**Supplement:**

**Li et al., Neural responses to reward in a gambling task: sex differences and individual variation in reward-driven impulsivity**

**Supplementary Figure S1.**

**
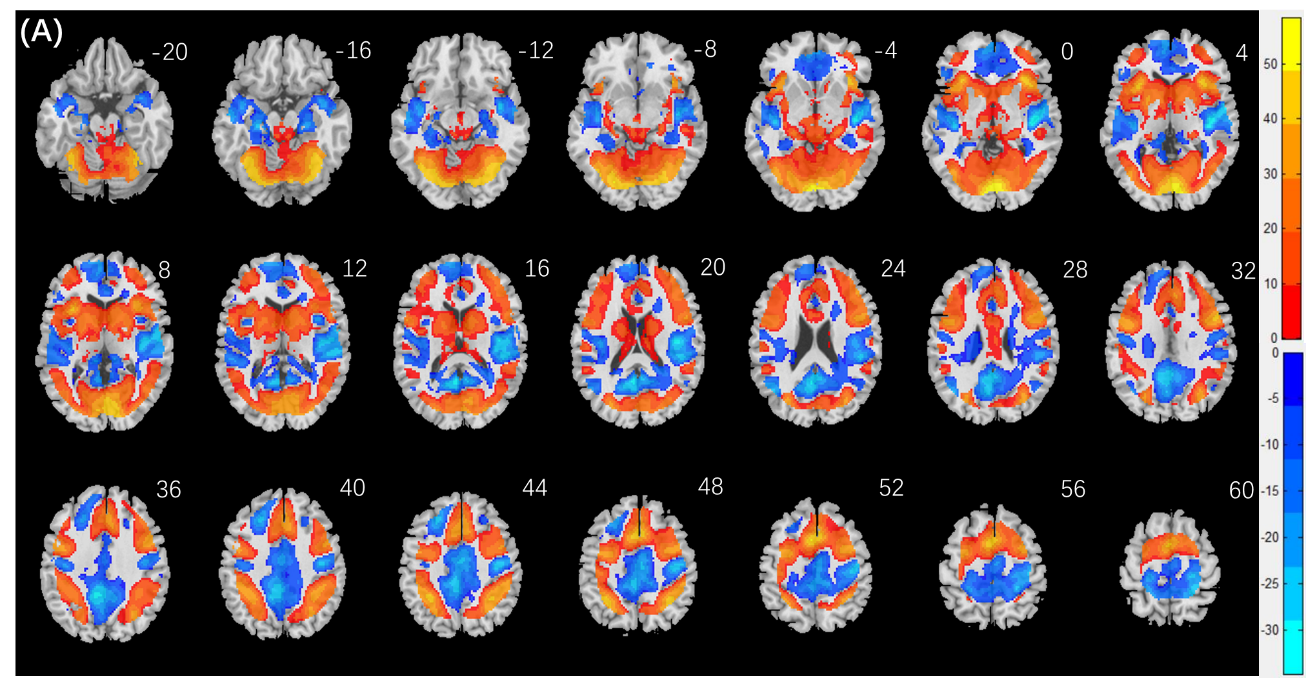
**

**
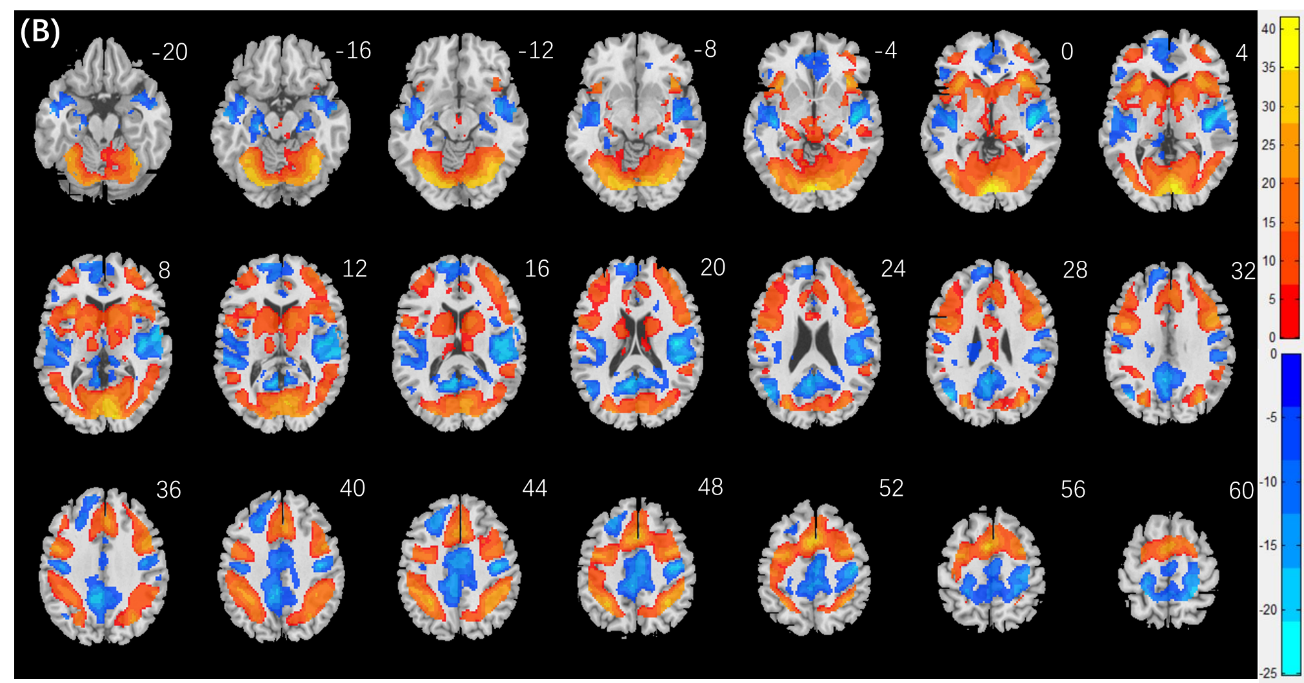
**

**
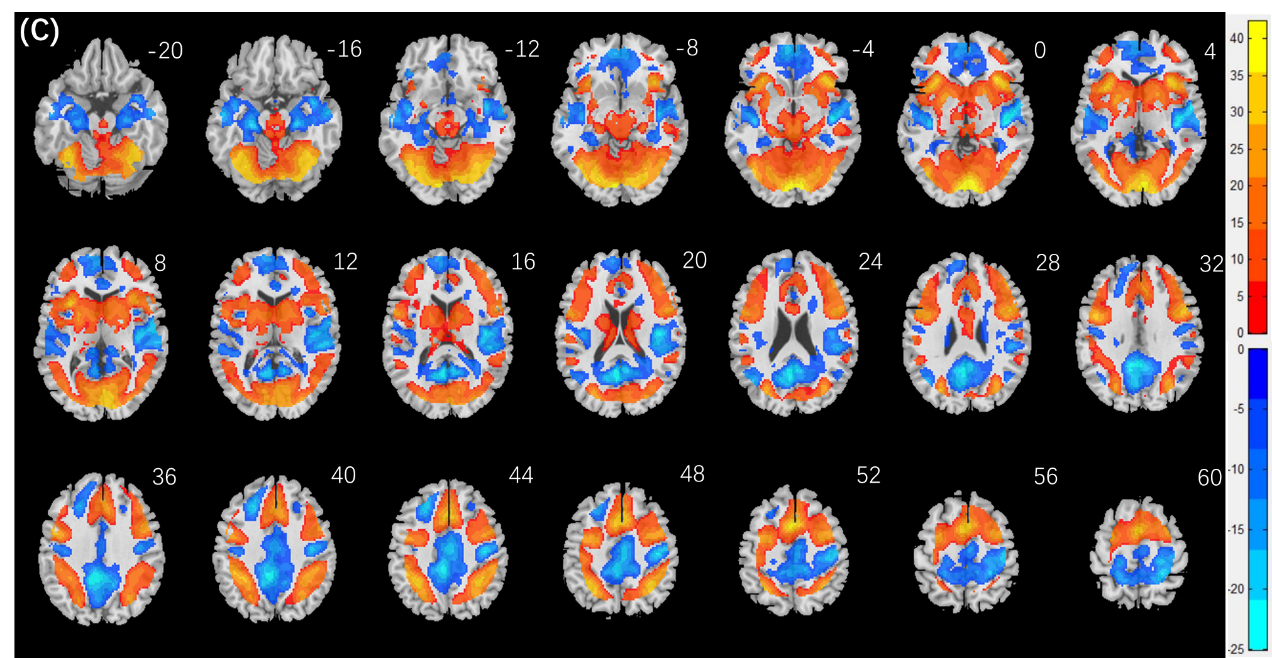
**

**Supplementary Figure S1.** One-sample t test of reward vs. baseline on **(A)** the entire cohort, **(B)** men, and **(C)** women. Color bars showed voxel T values. Warm color: reward > baseline; cool color: baseline > reward. Neurological orientation: right = right.

**Supplementary Figure S2.**

**
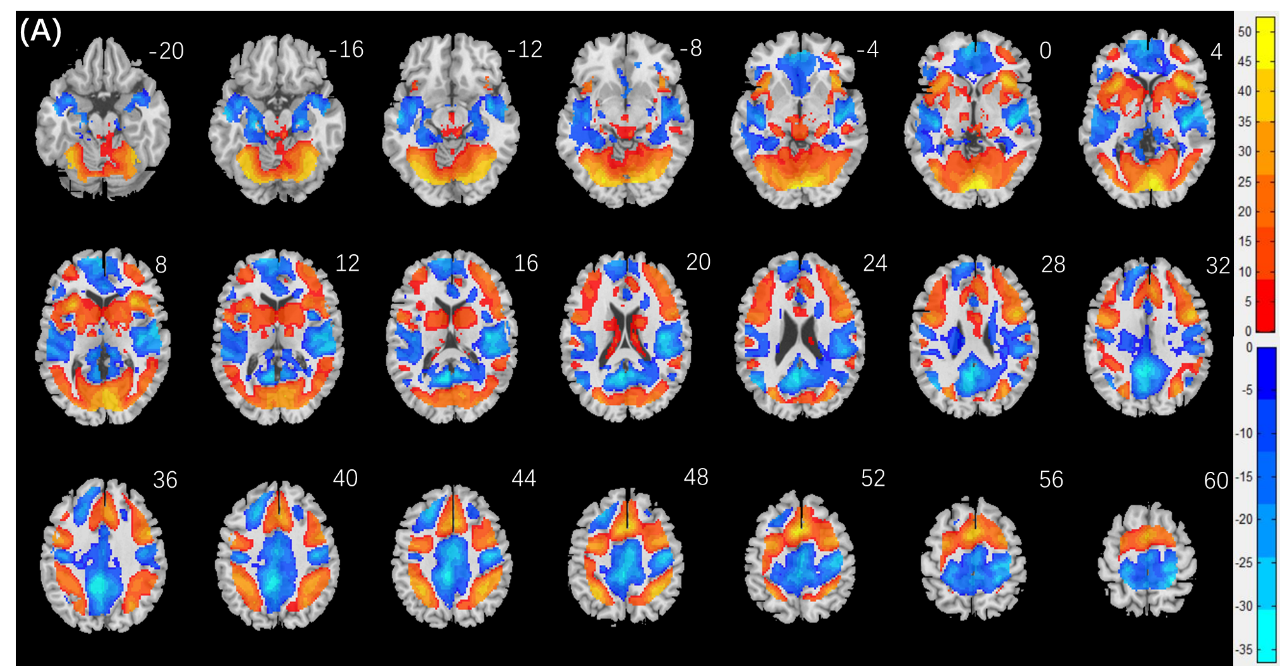
**

**
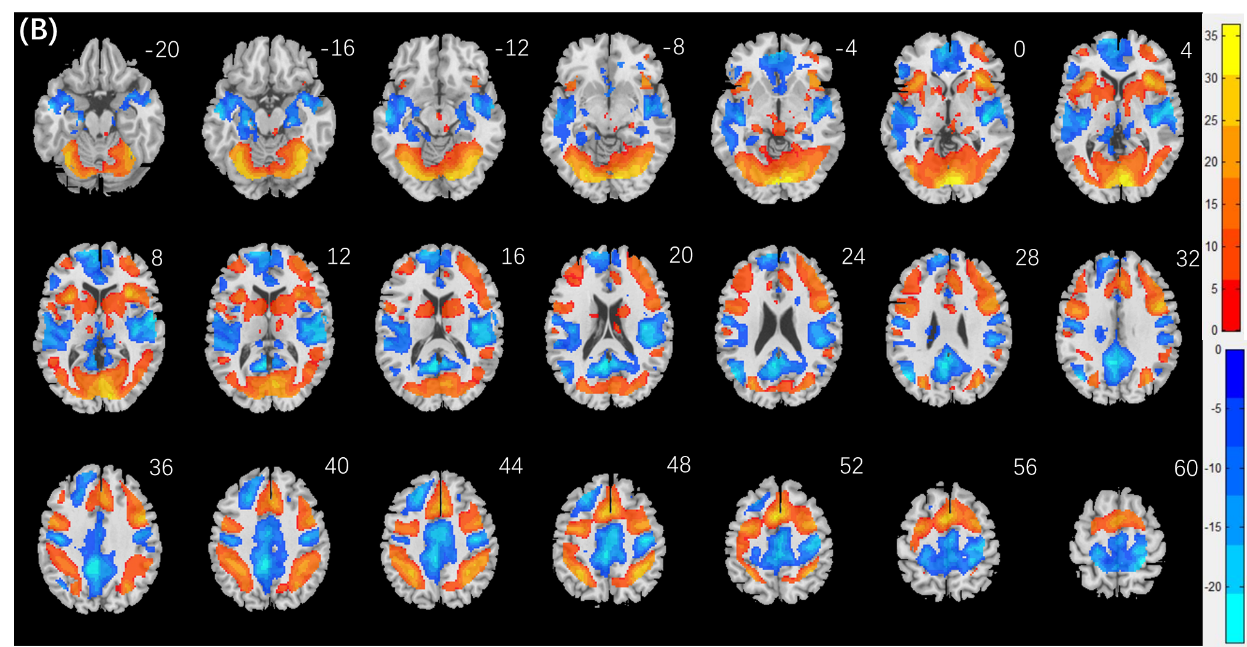
**

**
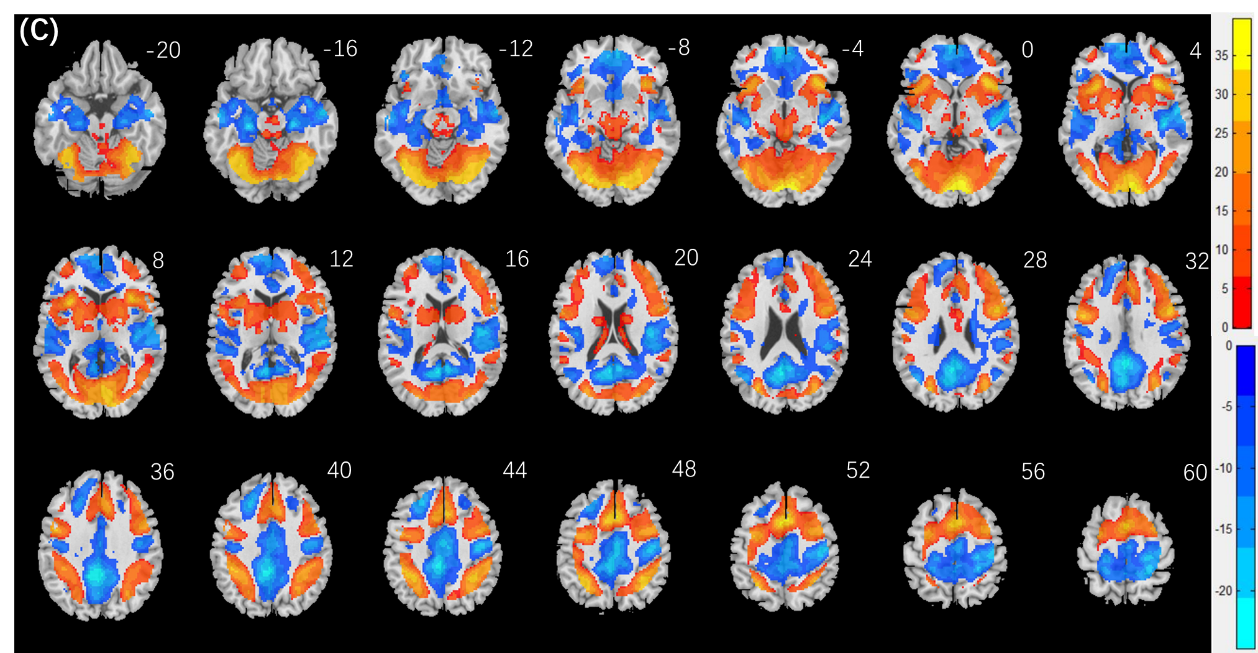
**

**Supplementary Figure S2.** One-sample t test of punishment vs. baseline on **(A)** the entire cohort, **(B)** men, and **(C)** women. Color bars showed voxel T values. Warm color: punishment > baseline; cool color: baseline > punishment. Neurological orientation: right = right.

**Supplementary Figure S3.**

**
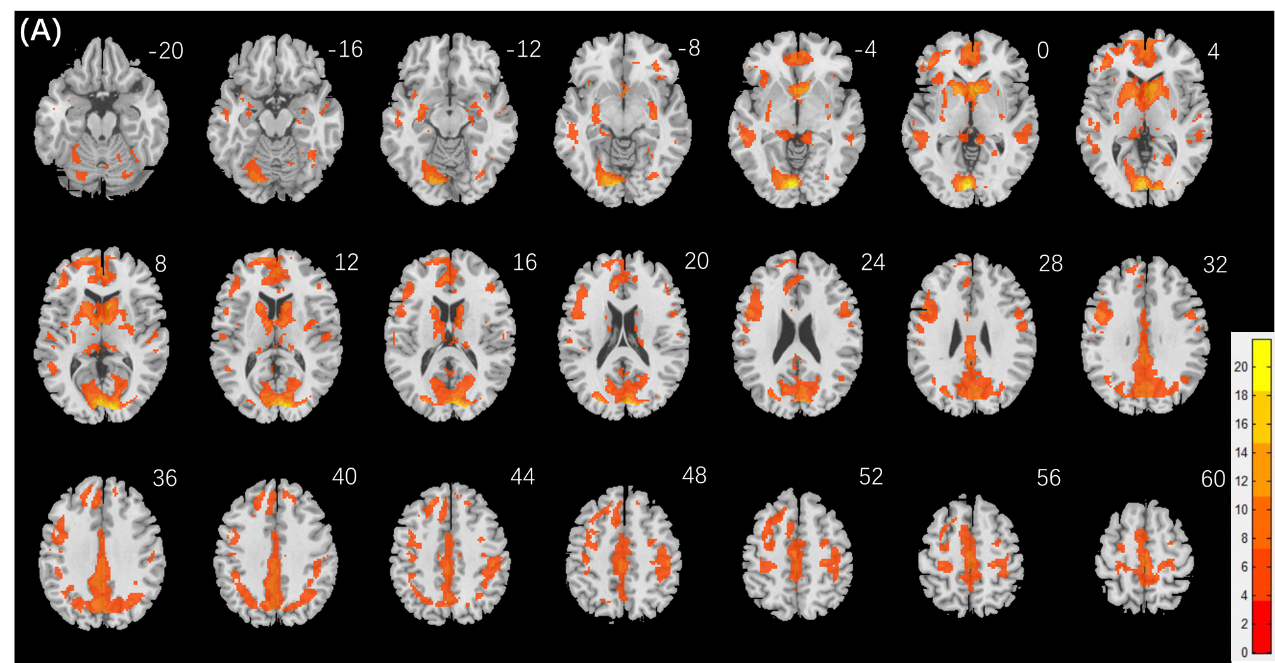
**

**
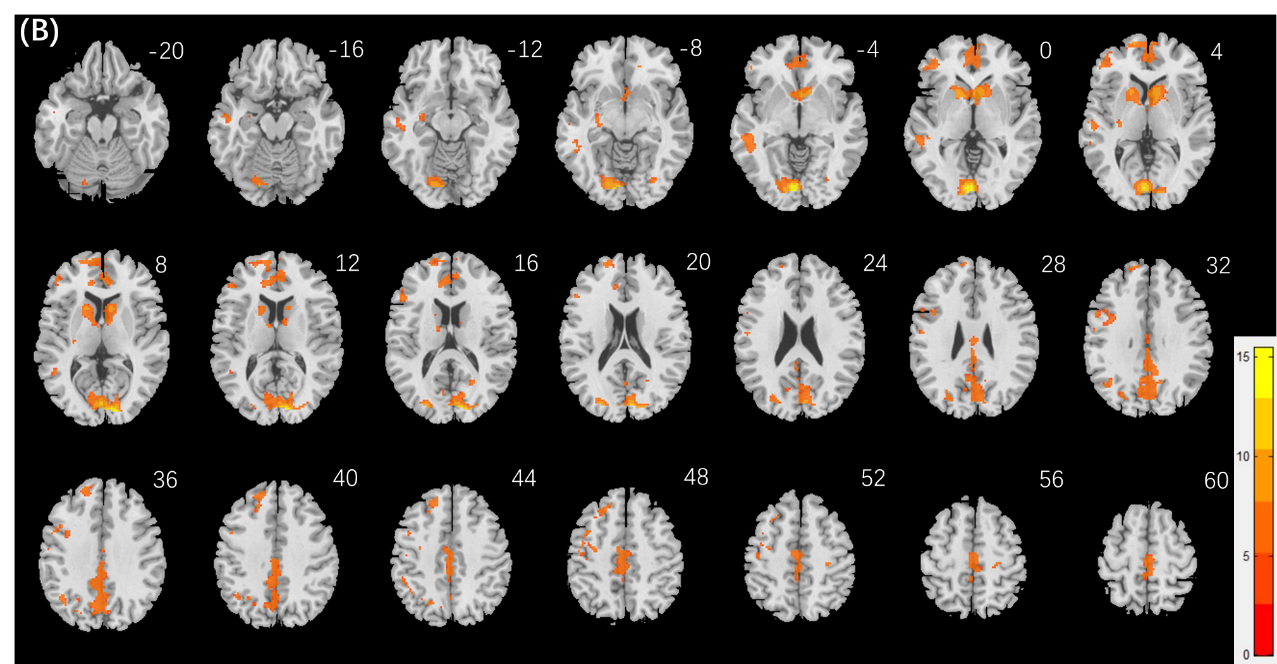
**

**
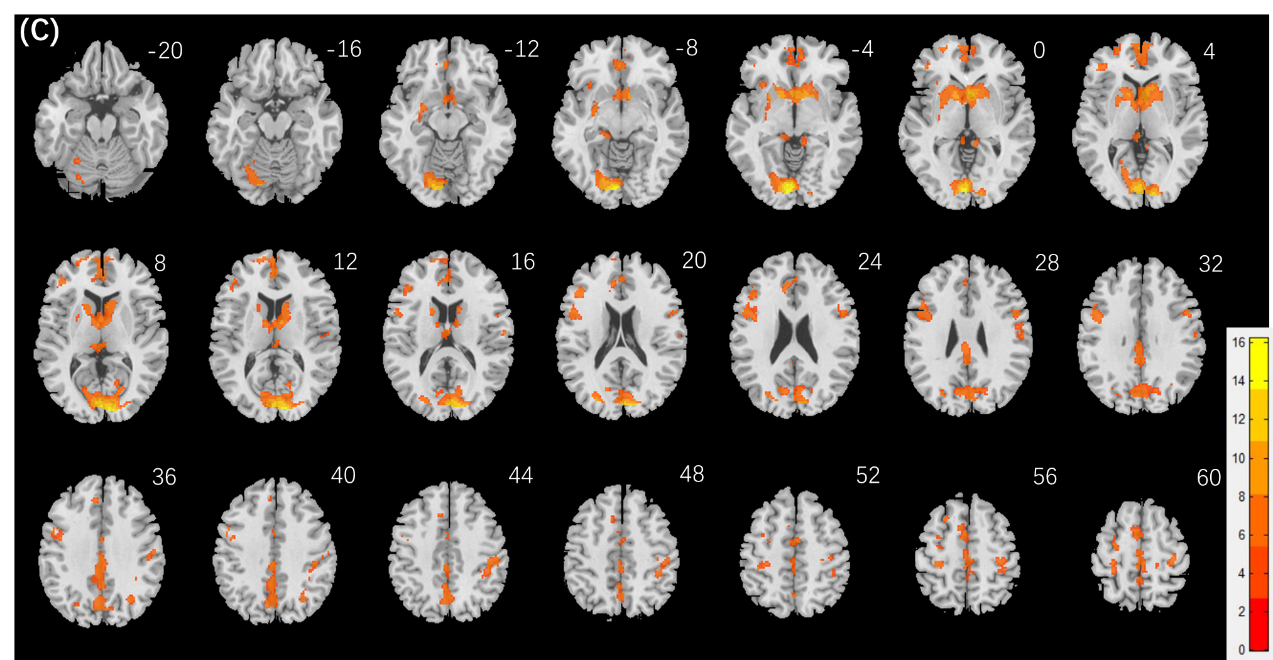
**

**Supplementary Figure S3.** One-sample t test of reward vs. punishment for **(A)** the entire cohort, **(B)** men, and **(C)** women. Color bars showed voxel T values. Warm color: reward > punishment; No clusters showing higher response to punishment than to reward; right = right.
